# Isobaric 4-Plex Tagging for Absolute Quantitation of Biological Acids in Diabetic Urine Using Capillary LC–MS/MS

**DOI:** 10.1021/acsmeasuresciau.1c00061

**Published:** 2022-03-03

**Authors:** Michael R. Armbruster, Scott F. Grady, Christopher K. Arnatt, James L. Edwards

**Affiliations:** Department of Chemistry and Biochemistry, Saint Louis University, 3501 Laclede Ave, St Louis, Missouri 63103, United States

**Keywords:** targeted metabolomics, isobaric labeling, multiplexing, diabetes, absolute quantitation

## Abstract

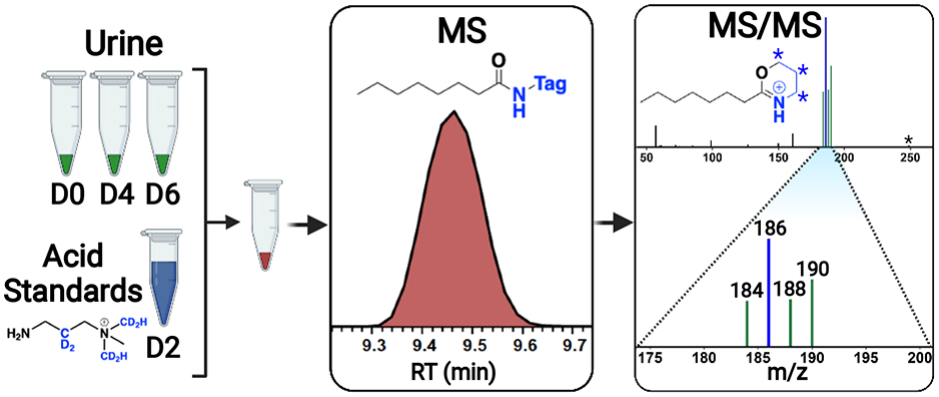

Isobaric labeling in mass spectrometry enables multiplexed absolute quantitation and high throughput, while minimizing full scan spectral complexity. Here, we use 4-plex isobaric labeling with a fixed positive charge tag to improve quantitation and throughput for polar carboxylic acid metabolites. The isobaric tag uses an isotope-encoded neutral loss to create mass-dependent reporters spaced 2 Da apart and was validated for both single- and double-tagged analytes. Tags were synthesized in-house using deuterated formaldehyde and methyl iodide in a total of four steps, producing cost-effective multiplexing. No chromatographic deuterium shifts were observed for single- or double-tagged analytes, producing consistent reporter ratios across each peak. Perfluoropentanoic acid was added to the sample to drastically increase retention of double-tagged analytes on a C18 column. Excess tag was scavenged and extracted using hexadecyl chloroformate after reaction completion. This allowed for removal of excess tag that typically causes ion suppression and column overloading. A total of 54 organic acids were investigated, producing an average linearity of 0.993, retention time relative standard deviation (RSD) of 0.58%, and intensity RSD of 12.1%. This method was used for absolute quantitation of acid metabolites comparing control and type 1 diabetic urine. Absolute quantitation of organic acids was achieved by using one isobaric lane for standards, thereby allowing for analysis of six urine samples in two injections. Quantified acids showed good agreement with previous work, and six significant changes were found. Overall, this method demonstrated 4-plex absolute quantitation of acids in a complex biological sample.

## Introduction

Absolute quantitation of metabolites across samples is hampered by ion suppression, instrument drift, and matrix effects.^1^ Chemical derivatization attaches a tag with properties beneficial for mass spectrometry (MS) analysis to a targeted functional group, often increasing ionization efficiency and throughput.^2^ While tagging improves limits of detection and quantitation, increased matrix effects and excess reagent can suppress ionization.^3^ Post-reaction cleanup steps (e.g., extractions) can mitigate these effects if there is an advantageous difference between the properties of the excess tag and the tagged analytes. For proteomics, a C18 or ion exchange column separates the tag from the tagged analytes.^4^ The hydrophobicity of small derivatized metabolites is often dominated by the tag, making these sample cleanup methods challenging in metabolomics.

Isotope tagging alleviates the problems of quantitation, throughput, and instrument drift. Isotope tagging methods are designed to create mass shifts between the same analyte across different samples. This mass shift can appear in the full scan (MS^1^) in the case of mass shift tagging or upon fragmentation (MS^2^) in the case of isobaric tagging. Isobaric labeling reduces spectral complexity by collapsing all tag variants to the same nominal mass in the MS^1^ scan.^5^ Each tag contains a balancer group, a reactive group, and a reporter group. The total number of isotopes across the balancer and reporter groups are equal for an isobaric set of tags. This causes co-isolation of all multiplexed samples and fragmentation of labeled analytes to produce unique, isotope-encoded reporters.^6^ The intensity of each reporter serves as an indicator of the relative amount of analyte in each sample.

**Figure 1:**
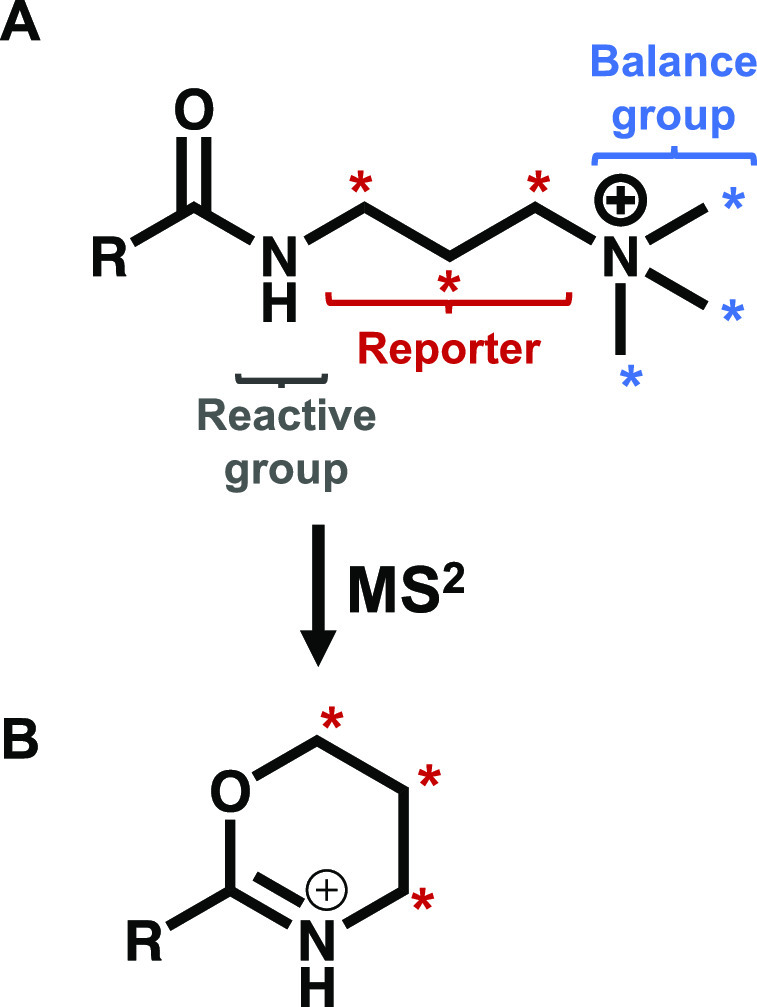
Trialkyl ammonium isobaric tagging. Example tagged analyte (A) and reporter formation (B) produced by the neutral loss of a trimethylamine. Asterisks indicate sites of D_2_ (red) or D_2_H (blue) incorporation.

While the *m/z* of reporters produced by fragmentation are often independent of the precursor ion (TMT,^7^ DiLeu,^8^ iTRAQ,^9^ and DiART^10^), other tags produce characteristic neutral losses.^11,12^ Pioneering work by the Reid group has proven the effectiveness of a stable cyclization to produce isotope-encoded reporters which remain attached to the analyte.^13−15^ A trialkylsulfonium or quaternary alkylammonium neutral loss provides a site for simple isotope manipulation of the balancer group,^16,17^ while the alkylamine chain allows for reporter isotopes to be synthetically incorporated (Figure 1).^18,19^ The energy requirements for fragmentation of the sulfonium group are lower than the quaternary amine in addition to being less dependent on analyte proton mobility.^16^ This low energy barrier makes the sulfonium better suited for proteomics than metabolomics because the cyclization must compete with many amide bond fragmentations.

Small polar metabolites rarely contain multiple amide bonds, which would reduce the penalty for using a quaternary amine over of a sulfonium moiety. Quaternary amines are synthetically desirable due to the simple attachment of isotopes using selective methylations of a primary amine with formaldehyde and/or methyl iodide.^20^ The deuterated forms of these two reagents are widely available and provide a cost-effective alternative to ^13^C and ^15^N labeling.^21^ These tags can be attached to metabolites using coupling reactions between a free amine on the tag and the acid group on metabolites.^22,23^ Most isobaric labeling workflows have been developed for proteomics experiments, with limited examples of metabolomic analysis.^10,24−28^ Differences in the hydrophobicity between peptides and polar metabolites require novel sample preparation workflows for tagging reactions.^29^

Here, a synthetic method and labeling workflow are presented for a set of four isobaric tags. The MS^2^ reporters are created through a neutral loss cyclization and are dependent on the tagged metabolite mass. Excess tag is scavenged and extracted to reduce ion suppression and column overloading, while a perfluoropentanoic acid sample modifier improves retention of double-tagged metabolites on a C18 column. This method is used for the multiplexed absolute quantitation of 37 acid metabolites in control and type 1 diabetic urine.

## Methods

### Standard Stock Creation

Metabolite standards were acquired from the MS metabolite library of standards (IROA Technologies, Sea Girt, NJ). Lactate, malate, succinate, fumarate, dimethyl glycine, benzoic acid, and 4-formylbenzoic acid (4-FBA) were purchased from Sigma Aldrich (St. Louis, MO). Each metabolite was reconstituted to 100 μM in 5% MeOH, mixed, dried, and reconstituted to create a 1 μM mixed acid stock in dimethylformamide (DMF). All other reagents were purchased from Sigma Aldrich unless otherwise noted.

### Tag Synthesis

#### Tert-butyl (3-aminopropyl)carbamate Isotopologues

**Scheme 1:**
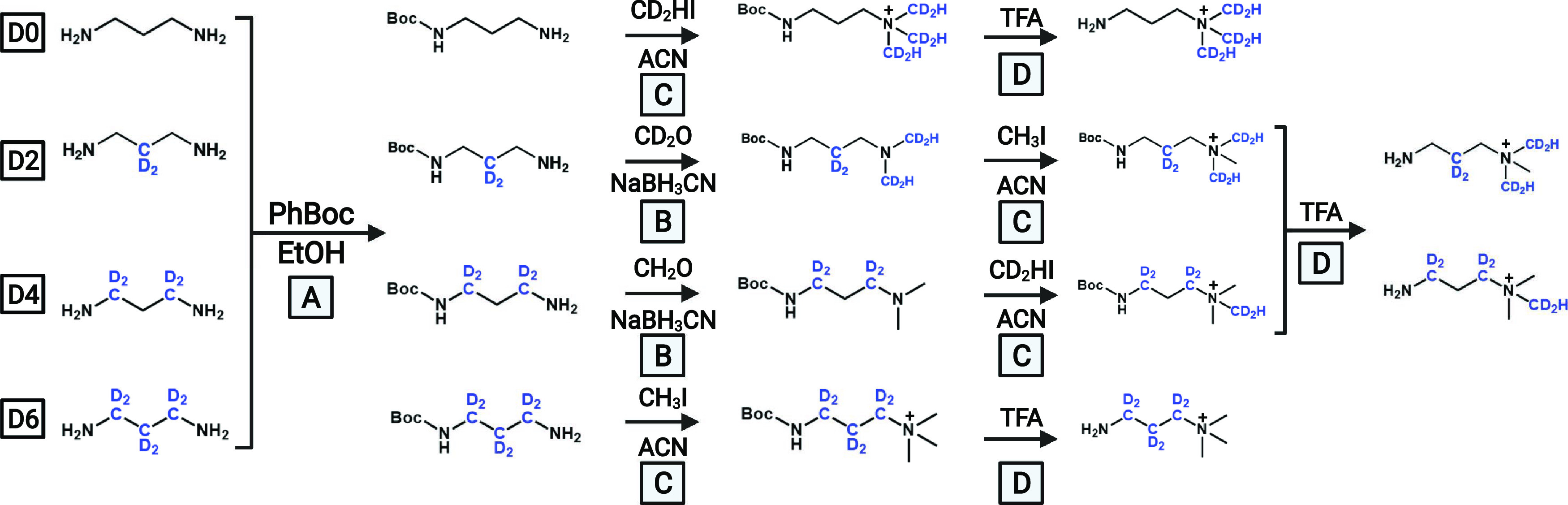
Synthetic Routes for Each of the 4 Isobaric Tags Used Isotopic variants of 1,3-diaminopropane were purchased as starting materials. (A) Starting materials were protected using tert-butyl phenyl carbonate (PhBoc). (B) Partial methylation of amines with reductive methylation. (C) Complete methylation of amines using iodomethane. (D) Boc deprotection in 4:1 DCM: trifluoroacetic acid

1,3-Diaminopropane (D_0_, D_2_, D_4_, or D_6_, CDN Isotopes, Point-Claire, Quebec, Canada) and tert-butyl phenyl carbonate were dissolved in ethanol, and the reaction mixture was refluxed overnight (Scheme 1). The reaction mixture was concentrated and reconstituted in water (25 mL), pH-adjusted to 3 with 2 M HCl, and washed with dichloromethane (DCM, 3× 40 mL). The aqueous phase pH was adjusted to 11 with 2 M NaOH and extracted with DCM in a separate collection flask. This organic layer was dried with Na_2_SO_4_ and concentrated to afford the product as a yellow oil (30% yield).

#### Tert-butyl (3-(dimethylamino)propyl)carbamate Isotopologues

tert-butyl (3-aminopropyl)carbamate (D_2_ or D_4_) was dissolved in acetonitrile, and 5 equivalents of formaldehyde (D_0_ or D_2_) (20% in H_2_O or D_2_O respectively) were added. 1.6 equivalents of sodium cyanoborohydride was added, and the reaction mixture was allowed to stir for 15 min before adjusting the pH to 7. The reaction mixture was stirred for 45 min, keeping the pH at 7 throughout. The mixture was then concentrated, and 4 mL of 2 M KOH was added and extracted with ether (3× 10 mL). The organic layer was washed once more with 4 mL of 0.5 M KOH, dried with Na_2_SO_4_, and concentrated to afford the product as a colorless oil (80% yield).

#### *3-*((Tert-butoxycarbonyl)amino)-N,N,N-trimethylpropan-1-aminium Isotopomers

tert-butyl (3-(dimethylamino)propyl)carbamate (D_4_ or D_6_) or tert-butyl (3-aminopropyl)carbamate (D_0_ or D_6_) was dissolved in acetonitrile in sealable, thick-walled reaction tubes, and potassium carbonate (4 eq) was added to each. To the D4 tert-butyl (3-(dimethylamino)propyl)carbamate and D_0_ tert-butyl (3-aminopropyl)carbamate, D_2_ iodomethane was added. To the D_6_ tert-butyl (3-(dimethylamino)propyl)carbamate and D6 tert-butyl (3-aminopropyl)carbamate, D_0_ iodomethane was added. The tubes were sealed, heated to 70 °C, and set to stir overnight. To each vial was added 2 mL of water, and the mixtures were stirred briefly. The top layers were removed by pipette and concentrated, then redissolved in DCM, and filtered to yield the corresponding methylated products (88% yield).

#### 3-Amino-N,N,N-trimethylpropan-1-aminium Isotopomers

The D_6_ 3-((tert-butoxycarbonyl)amino)-*N*,*N*,*N*-trimethylpropan-1-aminium isotopomers were dissolved in a 4:1 DCM:trifluoroacetic acid mixture and set to stir for 1 h, giving the desired products (100% yield). Each tag was observed in high isotopic purity using HRMS for exact mass analysis. All tags had an expected exact mass of 123.1763 Da and an observed *m/z* of 123.1764 (Figure S1). The arrangement of deuterium atoms on each tag was verified by proton NMR (Figure S2) and by tagging sarcosine and fragmenting to observe the corresponding reporter ions (Figure S3). Expected exact masses for the D0, D2, D4, and D6 reporters of sarcosine are 129.10, 131.11, 133.13, and 135.14 Da, respectively. Observed *m/z* values for the respective reporters were 129.10, 131.11, 133.13, and 135.14.

### Liquid Chromatography

LCMS grade water and acetonitrile were purchased from Fisher Scientific (Pittsburgh, PA). Capillary columns with photopolymerized integrated frits and emitter tips were fabricated in-house from fused silica capillary (Polymicro Technologies, Phoenix, AZ) with dimensions 17.5 cm × 50 μm and packed with 3 μm Atlantis T3 C18 particles (Milford, MA) as previously described.^30^ Flow through the column was delivered by a Thermo Vanquish (Thermo Fisher Scientific, Waltham, MA) LC pump and autosampler connected to a stainless steel tee which acted as a flow splitter. The split was a 50 μm × 100 cm open capillary. The column was operated at a flow rate of 125 nL/min with an injection of 4 nL split from the bulk flow of 175 μL/min and injection of 6 μL. The flow rate was determined based on the experimental dead time and used to determine a split ratio of 1:1400. Mobile phase A was 0.1% formic acid in water. Mobile phase B was 0.1% formic acid in acetonitrile. The gradient was as follows: 0–1 min, 0% B; 12 min, 98% B; 14 min, 98% B; 14.1–25 min, 0% B.

### Creatinine Normalization

Urine was obtained from Lee Biosolutions (Maryland Heights, MO) as single donor samples. All raw urine samples were centrifuged at 21,000 *×g* for 10 min, and the supernatant was used for further analysis. Creatinine concentration was determined before performing the chemical tagging reaction for each urine sample using D_3_-labeled creatinine based on previous methods.^31^ Urine samples were diluted 1000× and spiked to 9 μg/mL with D_3_ creatinine. Separations of creatinine were performed on the same C18 capillary column as described for isobaric analysis. A parallel reaction monitoring (PRM) method was used to monitor the transitions of biological creatinine from *m/z* 114 to 86 and D_3_-labeled creatinine standard from *m/z* 117 to 89 (Figure S4).

### Derivatization

**Figure 2:**
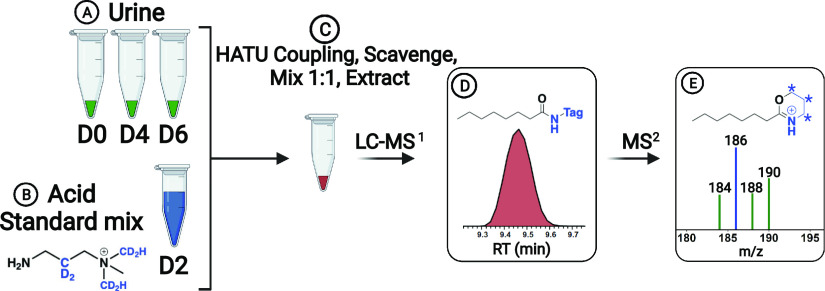
Schematic of the analytical workflow for acid quantitation. Biological urine samples are dried, reconstituted into DMF, and then aliquoted into vials containing isobaric tags D0, D4, and D6 (A). Separately, 54 standards are mixed into a vial containing tag D2 (B). These four vials are individually coupled using HATU, excess tag scavenged, then mixed, and extracted as described in the text (C). This mixed sample is dried, reconstituted, and analyzed by LC–MS/MS (D). Detection of a peak triggers a MS^2^ fragmentation event, which produces four characteristic neutral losses (E). Asterisks indicate sites of possible deuterium incorporation on the reporter ion.

Urine was diluted with LC–MS water to match all creatinine concentrations as determined by isotope analysis (Table S1). All samples were diluted to 277 μg/mL creatinine and spiked with 1 μM of 4-FBA. Ten microliters of each sample was dried by vacuum centrifugation and reconstituted in 100 μL of DMF. Four microliters of one of the isobaric tag stocks (250 mM) was added to each vial, followed by 1 μL of 500 mM HATU and HOBt with 2 μL of triethylamine. Separately, mixed standards were reacted with isobaric tag D2 (Scheme 1 and Figure 2). The reaction was shaken at room temperature for 70 min as previously described.^32^ Two microliters of hexadecyl chloroformate was added to the vials to scavenge excess tag and biological primary amines. The samples were then mixed and spiked with 1 μM D2-tagged standards, as shown in Figure 2. The tagged analytes were Folch extracted by adding 2 eq of 2:1 CHCl_3_:MeOH, then 0.3 eq water to induce phase separation. The aqueous phase was removed and dried in a vacuum centrifuge and then reconstituted to 10 μL with water containing 0.1% formic acid and 30 mM perfluoropentanoic acid (PFPeA).

### MS Analysis

All samples were analyzed on a Q-Exactive Orbitrap (Thermo Fisher Scientific, Waltham, MA) coupled to a nanospray flex ion source operating in positive ionization mode. Spray voltage was set to 1.75 kV and capillary temperature to 200 °C. MS fragmentation runs were performed with an inclusion list at MS^1^ resolution of 35 K and automatic gain control (AGC) of 1e^6^ with a maximum injection time of 50 ms and a scan range of 140–800 *m/z*. The top 5 peaks triggered MS^2^ events at a resolution of 17.5 K, AGC of 1e^5^, maximum injection time of 50 ms, isolation of 0.7 *m/z*, dynamic exclusion of 5 s, and normalized collision induced dissociation energy (nCID) of 35. Reporter consistency across each peak was determined by a PRM method with the same resolution, AGC, fragmentation energy, and isolation width as the data-dependent method.

### Data Analysis

All data analyses were performed in R (version 4.0.5). Thermo .raw files were converted to .mzXML using MSConvert.^33^ The findNL function from CluMSID was used to search for the expected isotope-encoded neutral losses using a 10 ppm mass window.^34^ The intensity ratios between each biological sample and the internal standard reporter were taken at the peak of each precursor for quantitation. All concentrations in urine are reported as μM/mmol creatinine, and significant differences were determined using a two-tailed *t* test with *p* < 0.05 indicating significance.

## Results and Discussion

The tag structure employs simple synthetic routes and cost-efficient reagents to produce four isobaric variants (Scheme 1). The quaternary amine serves as a site for isotope manipulation and provides a fixed positive charge to enhance signal response in positive mode analysis. High reporter isotope purity was maintained by minimizing the synthetic steps to complete synthesis (Figure S3). The addition of many synthetic precursors with isotopic purity <100% would have produced degenerate signals. This reduces signals attributed to the expected tagged analyte and complicates data analysis.

**Figure 3:**
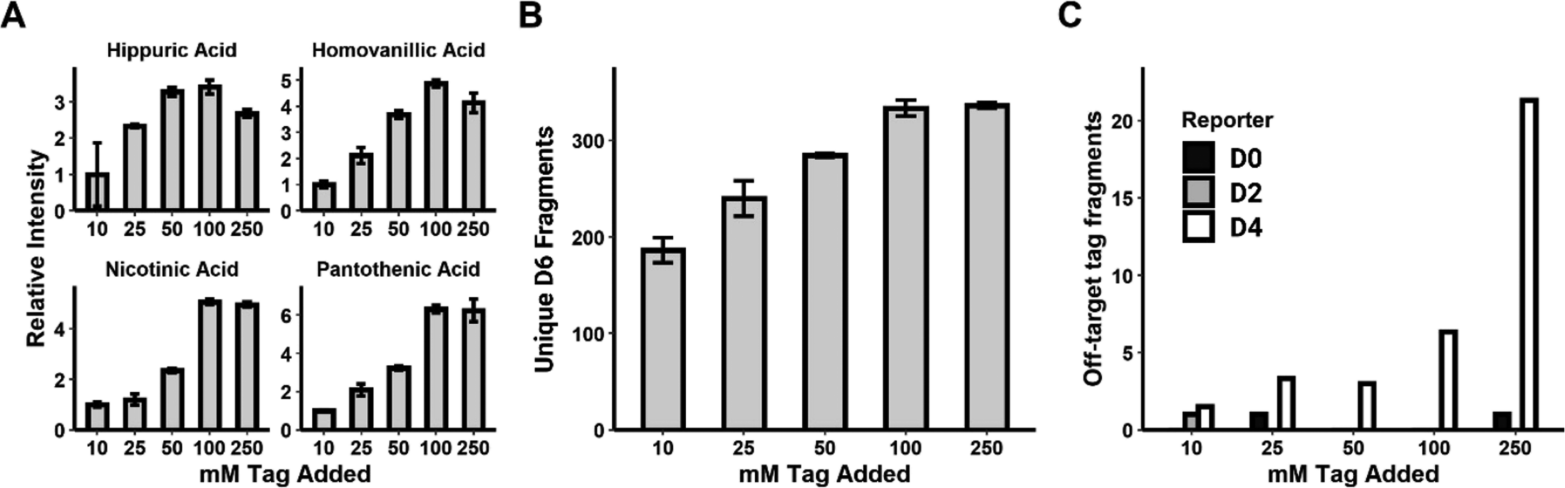
Reaction optimization was performed on pooled urine by ramping tag D6 concentration using a DDA method (*n* = 3). MS^1^ analyte intensities are shown relative to the mean at 10 mM tag added. There are no statistically significant increases in MS^1^ analyte peak heights (A) or number of unique parent masses producing the D6 reporter (B) between 100 and 250 mM tag added. Additionally, minimal false hits are observed for the other three tags not used in this experiment at 100 mM tag (C). Error bars are shown as ± standard deviation.

While coupling reactions have been optimized for fatty acid derivatization in nonpolar solvents with similar tags,^17,23^ the primary amine reactive group must outcompete biological amines when extracting polar solvents. The reaction was investigated in pooled urine to determine the concentration of tag required to produce reaction completion. A data-dependent analysis method (DDA) was used to monitor both analyte intensity and the number of precursor ions which produced the expected neutral loss. Both factors reached a maximum at 100 mM of tag in solution (Figure 3A,B). Reporters from the other three tags were searched to assess isotope overlap in a complex sample. The number of neutral losses attributed to tags that were not added was minimal, indicating high reporter purity (Figure 3C). These data confirm that the reaction is complete, with minimal isotope reporter overlap.

**Figure 4:**
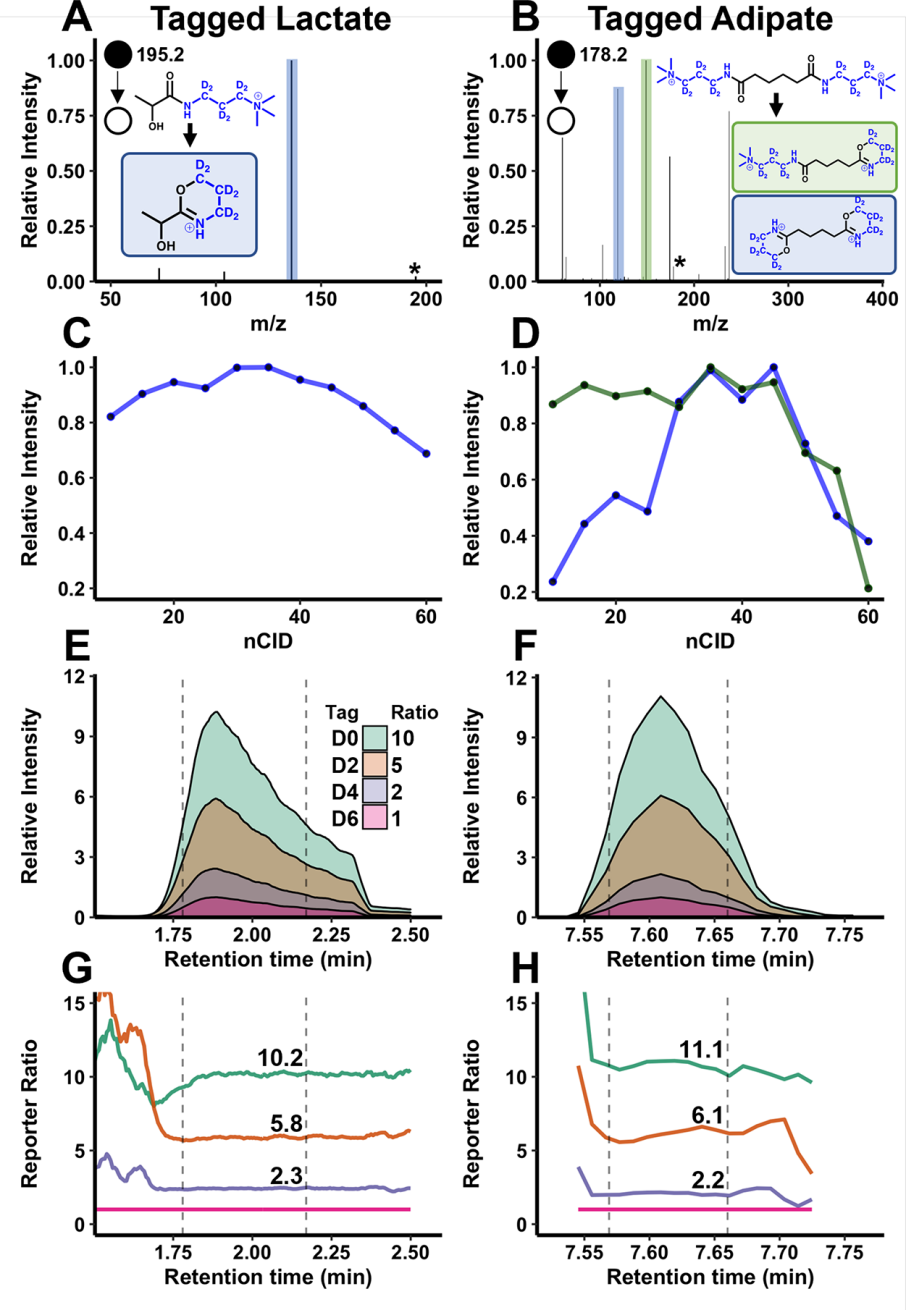
Fragmentation of D6-tagged valerate (A) and adipate (B) produce strong reporter ions. Reporters formed by complete ring formation are highlighted in blue, while the single ring formation for double-tagged adipate is highlighted in green. Asterisks indicate the precursor ion. Fragment optimization was performed to determine optimum fragmentation energy across a range of tagged compounds (C, D). Single ring formation is shown in green for analytes which are double-tagged. Minimal deuterium shifts are observed across the peak for each tag version (E, F). The lack of deuterium shifts results in consistent reporter intensity across the top 50% of each peak, shown with dashed vertical lines (G, H). Reporters from complete cyclization are shown for adipate. The average experimental ratio to D6 across the top 50% of each peak is shown in (G and H).

Neutral loss of the trimethylamine group produced efficient reporters for most analytes (Figure 4A). Analyte-dependent fragmentation was observed for some metabolites with multiple amide bonds or tags attached (Figure 4B). Double-tagged analytes produce a mix of single and double ring formation. This produces two sets of reporters: one from neutral losses of 59–65 (double ring formation) and one from 29.5 to 32.5 (single ring formation). All neutral losses show similar ratios (Figure S5), which is consistent with reports of multiple reporter quantitation in proteomics.^35^ For pantothenate, we observe multiple isotope-encoded reporters (Figure S6) due to competing fragmentation from the native amide bond and nearby mobile proton. Despite this mixed fragmentation, the reporter is observed at an acceptable intensity for quantitation (relative standard deviation, RSD = 4.8%, *R*^2^ = 0.997).

Collision energy optimization found an nCID value of 35% produced acceptable reporter intensities across a range of analytes (Figure 4C,D). Incorporation of deuterium is often avoided due to the potential for retention time shifts across tags. This is caused by the increase in polarity of deuterium compared to hydrogen, which causes earlier elution on reverse phase columns.^36^ Previous work has shown that incorporation of the deuterium around a quaternary amine drastically reduces retention time shifts, allowing for the synthesis of cost-efficient tags.^37^ Similarly, retention time shifts from these tags are not observed (Figure 4E,F). This provides consistent reporter ratios across each peak for tagged analytes mixed 10:5:2:1 (Figure 4G,H). The average ratio variance of the D0, D2, and D4 reporters across the top 50% of each peak was 1.6% for lactate and 3.9% for adipate. This indicates strong quantitative potential of this system.

**Figure 5:**
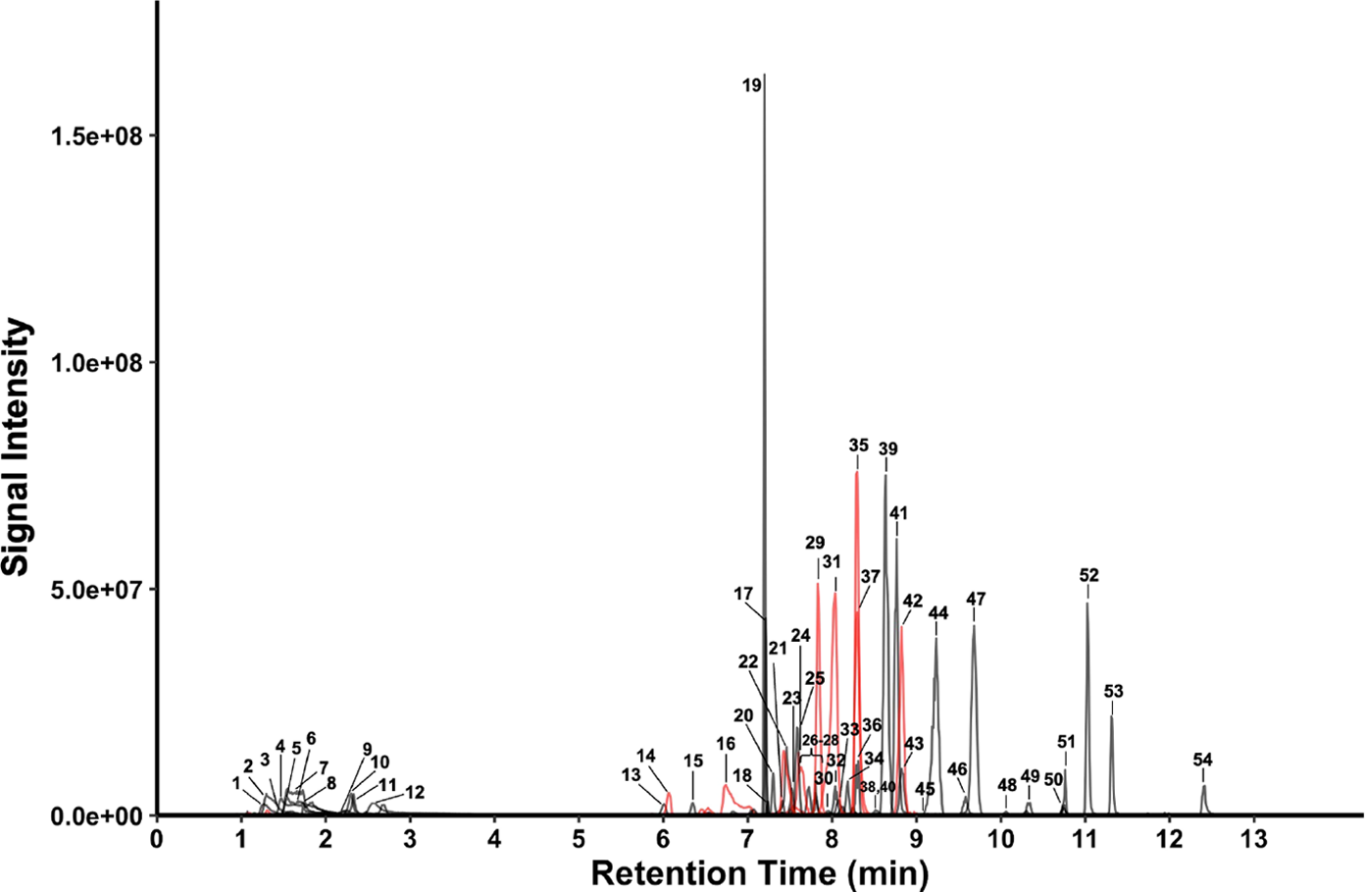
Extracted ion chromatogram of 54 tagged organic acid analyte standards. PFPeA was added to the sample at 30 mM to improve retention of tagged analytes. Red peaks show double-tagged analytes. Numbered peaks refer to the ID column in Table 1.

Separation of tagged analytes on a reverse phase column was hampered by the extreme polarity of the quaternary amine tag. We have previously shown the ability of PFPeA to aid in the retention of quaternary amine tagged compounds.^37^ PFPeA was added to the sample vial at a higher concentration (30 mM) in contrast to its previous use as a mobile phase additive. This resulted in dramatically increased retention of double-tagged acids (Table 1 and Figure 5), while using minimal PFPeA to avoid introduction of excess perfluorinated acids into the environment. Of note, separation and isolation of monomethylgutarate from its isomer 2-methylgutarate is aided by our tagging scheme. Monomethylglutarate is singly tagged, resulting in better retention and a larger *m/z* (10.8 min, 251 *m/z*) compared to double-tagged 2-methylglutarate (7.5 min, 178 *m/z*). Reaction and extraction of the excess tag using hexadecyl chloroformate improved analyte intensity by 23% on average (data not shown), and minimal hexadecyl-tag is observed in the extracted samples (Figure S7). While a small amount of unreacted tag (*m/z* 123.2) is observed, it is typically excluded by the quadrupole to minimize nonproductive charges entering the C-trap in both MS^1^ and MS^2^ scans.

**Table 1:** Investigated Analytes, Retention Time, Linearity, and Relative Standard Deviation of Standards

ID	analyte	*R*^2^ (*n* = 4)	RT (min)	intersample RT RSD (*n* = 3) (%)	intrasample signal response RSD (*n* = 4) (%)
1	glyceric acid	1.000	1.3	0.18	8.5
2	gluconate	0.999	1.3	0.11	23.0
3	N-acetylasparagine	0.999	1.5	0.37	7.7
4	N-acetylglycine	1.000	1.5	0.15	10.8
5	glyoxylic acid	0.990	1.5	0.23	20.3
6	3-phosphoglyceric acid	0.975	1.6	0.08	8.7
7	ureidopropionic acid	0.987	1.6	0.62	4.7
8	lactate	0.999	1.6	0.31	14.4
9	acetoacetate	0.985	2.1	0.64	22.9
10	2-oxobutyric acid	0.958	2.3	4.79	17.6
11	N-acetylalanine	0.999	2.3	0.23	10.6
12	2-hydroxybutyric acid	1.000	2.3	0.68	14.3
13	4-imidazoleacetic acid	1.000	6.0	2.19	11.3
14	malate	1.000	6.1	0.08	18.8
15	dimethyl Glycine	0.983	6.4	0.37	4.1
16	N-acetylglutamic acid	0.985	6.7	0.43	16.7
17	sarcosine	0.980	7.2	0.68	21.7
18	orotic acid	0.997	7.2	0.12	15.2
19	4-acetamidobutanoic acid	1.000	7.2	0.02	6.0
20	nicotinic acid	0.996	7.3	0.08	2.2
21	butyric acid/Isobutyric acid	0.998	7.4	0.83	2.2
22	N-acetylproline	0.999	7.5	1.65	11.7
23	4-imidazoleacrylic acid	0.995	7.5	3.25	10.7
24	2-methylglutaric acid	0.987	7.5	4.96	18.3
25	pantothenic acid	0.997	7.6	0.15	4.8
26	adipic acid	0.999	7.6	0.30	21.4
27	mevalonic acid	0.990	7.7	0.09	30.6
28	N-acetylserine	0.956	7.8	0.14	6.3
29	pimelic acid	1.000	7.8	0.09	15.7
30	4-hydroxybenzoic acid	0.999	8.0	0.13	19.7
31	suberic acid	0.999	8.0	0.14	12.5
32	hydroxyphenyllactic acid	0.998	8.1	0.17	24.0
33	3-hydroxyphenylacetic acid	1.000	8.1	0.19	13.1
34	mandelic acid	0.997	8.3	0.29	7.6
35	azelaic acid	0.998	8.3	0.18	3.9
36	*p*-hydroxyphenylacetic acid	0.999	8.3	0.29	17.9
37	fumarate	0.998	8.4	0.24	7.0
38	valeric acid	1.000	8.5	0.15	22.5
39	hippuric acid	0.996	8.6	0.24	4.5
40	N-acetyl-methionine	0.999	8.6	0.20	7.0
41	N-acetylleucine/isoleucine	0.987	8.8	0.13	12.5
42	sebacic acid	0.992	8.8	0.24	12.2
43	benzoic acid	1.000	8.8	0.25	3.5
44	*N*-acetylphenylalanine	0.995	9.2	0.17	11.6
45	α-lipoic acid	0.998	9.3	3.32	5.3
46	caproic acid	0.995	9.6	0.19	7.8
47	3-methyl-2-oxovaleric acid	0.999	9.7	0.09	12.7
48	3,4-dihydroxyphenylacetic acid	0.955	10.1	0.23	7.5
49	valproic acid	0.965	10.2	0.03	14.2
50	monomethyl glutaric acid	0.999	10.8	0.09	13.3
51	caprylic acid	0.998	10.8	0.03	4.0
52	pelargonic acid	1.000	11.0	0.09	7.1
53	capric acid	0.999	11.3	0.10	9.0
54	myristic acid	0.999	12.4	0.06	12.7
	**average**	**0.993**	**7.0**	**0.58**	**12.1**

Tagged acids were mixed 1:2:5:10 (500 nM to 5 μM) to assess the analytical performance of the developed method. Mixing ratios were repeated four times with varying concentrations attributed to each tag to ensure that analytical performance was not dependent on the isotopic variant of the tag. A linear response was observed across an order of magnitude, with an average linearity of 0.993 (Table 1). Samples were mixed 1:1:1:1 to determine the reproducibility across each isotope lane and produced an average signal intensity RSD of 12%. Additional sample handling and reactions with differentially synthesized tags can reduce reproducibility, but this is often recovered by improvements to quantitation and reduced batch effects. Intersample retention time repeatability was determined by triplicate injections of pooled urine tagged and spiked with a 1 μM analyte mix. Retention times across injections were extremely reproducible with an average RSD of 0.58%. These consistent retention times, in addition to the 2 Da reporter spacing, could enable robust MRM analysis on triple quadrupole systems with scheduled retention time windows for improved sensitivity. Taken as a whole, this method allows for the absolute quantitation of organic acids.

This workflow was used to quantitate changes of acid metabolites in control and type 1 diabetic urine. All urine was normalized to creatinine prior to tagging, and quantitation was performed using the isotope ratio of a spiked standard reporter. Acquiring many untagged, isotopic metabolites is often unfeasible due to price and availability constraints. Here, all 54 nonisotopic standards were reacted with an isobaric tag, providing an isotope-encoded standard peak for every analyte. This large standard set minimizes sample to sample variation by accounting for matrix effects and instrument drift across injections. Quantitative data for 6 biological samples were obtained in two injections in 50 min of instrument time. All acid concentrations were compared to values from the human urine metabolome database,^38^ with all but dimethyl glycine showing good agreement. A complete view of the quantified acids is presented in Table S2.

**Figure 6:**
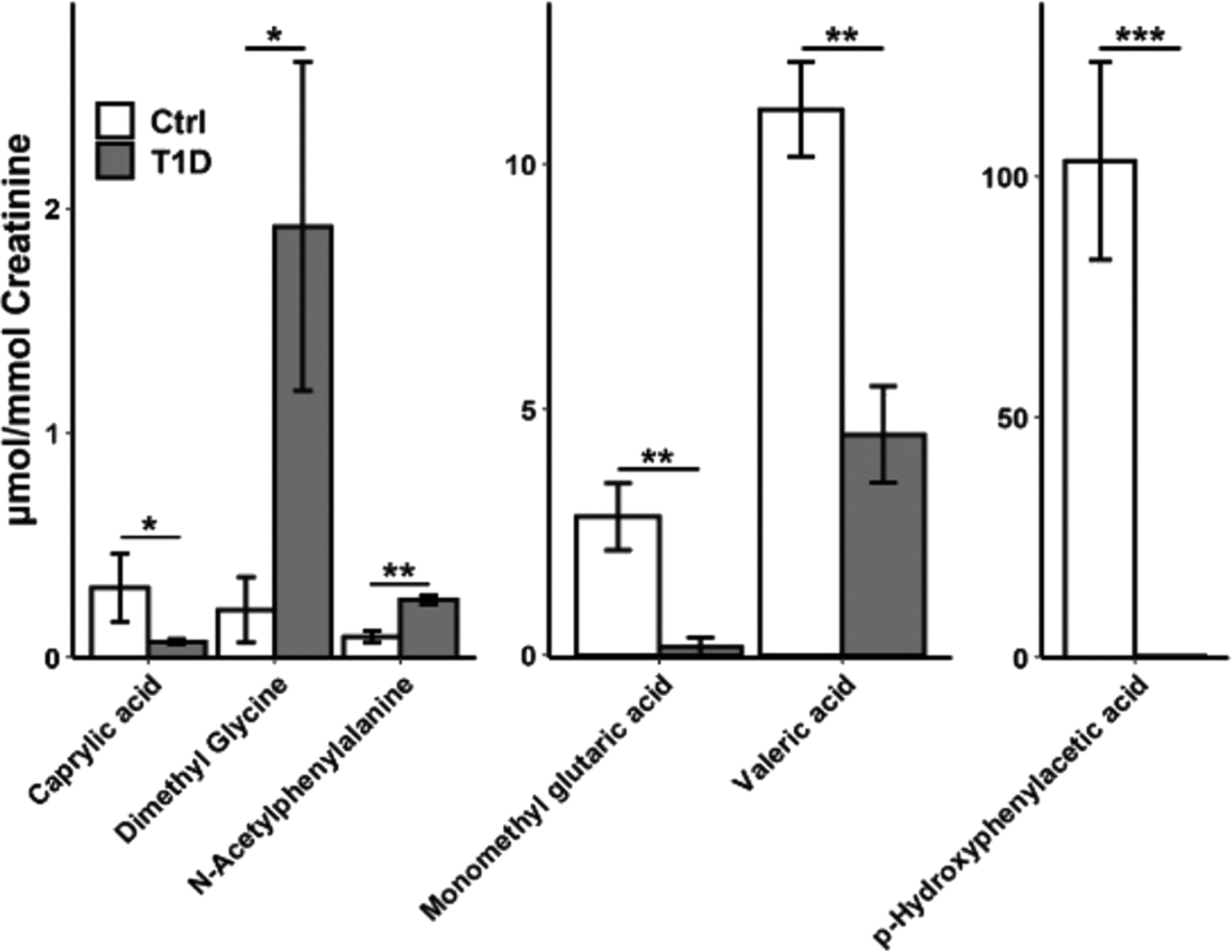
Statistically significant changes observed between control and T1D urine (*n* = 3). Error bars are shown as ± standard deviation. * = *p* < 0.05, ** = *p* < 0.01, *** = *p* < 0.001.

Significant changes in six acids were observed from diabetic urine (Figure 6). Multiple metabolites related to glomerular filtration rate are significantly altered, including dimethylglycine^39^ and *N*-acetylphenylalanine.^40^ In addition, decreases in medium chain fatty acids are associated with altered lipid metabolism.^41^*P*-hydroxyphenylacetic acid produced a large fold change outside our validated linearity and is considered to be largely dependent on gut microbiota and diet,^42^ requiring further investigation. To this end, further study on the microbiome in diabetes is warranted. Monomethylglutaric acid was detected at similar concentrations as previous studies,^38^ but has not been widely linked to diabetes.

## Conclusions

Here, we presented a synthetic route and LC–MS method for the 4-plex absolute quantitation of polar metabolites in urine. Each of the four tags was synthesized in minimal steps, producing isotopically pure reporters. Mindful tag design neutralized retention time shifts from deuterium incorporation, producing excellent quantitation. While some analyte-dependent fragmentation was observed for metabolites with a native amide bond, reporter generation was sufficient for quantitation. Retention of the polar, double-tagged analytes was drastically improved by adding PFPeA to the sample. Thirty-seven metabolites were quantified across six samples in under 50 min of instrument time. Six significant changes were observed, and quantified metabolites agreed well with previously published work.

While isobaric labeling provides many benefits to throughput and quantitation, its adoption has been limited for metabolomics workflows. This may be due to the stochastic nature of data-dependent methods, limited MS^2^ structural information, or simply a lack of cost-efficient methods. The presented tags provide a template for extremely cost-efficient multiplexing, but minimal metabolite identification in the MS^2^ spectra due to their efficient reporter formation. These qualities made it ideal for targeted, high throughput methods. While the presented tags allow for simultaneous analysis of up to four samples, further ^13^C or ^15^N incorporation could produce additional reporters for higher levels of multiplexing on both low- and high-resolution instruments. Isotope modification around the quaternary amine is trivial, as commercially available variants of formaldehyde and methyl iodide are widespread. Additional synthesis or coupling reactions could also expand the number of organic acids and targeted functional groups to cover a wide range of metabolites and account for the structural heterogeneity of the metabolome.

## Supporting Information Available

Characterization of the synthesized tags by LC-HRMS and NMR; HRMS^2^ of tagged sarcosine, creatinine, tagged adipic acid, and tagged pantothenic acid; hexadecyl-tag extraction efficiency; creatinine analysis results, and all quantified acids in control and T1D urine (PDF)
