# Supplementary material for: Isobaric 4-Plex Tagging for Absolute Quantitation of Biological Acids in Diabetic Urine Using Capillary LC–MS/MS
Source: ACS Meas Sci Au. 2022 Mar 3;2(3):287–95. doi: 10.1021/acsmeasuresciau.1c00061 (PMC9204807; doi:10.1021/acsmeasuresciau.1c00061)
Supplement: Supplementary file 1 — tg1c00061_si_001.pdf [file tg1c00061_si_001.pdf]

Supplementary Material for:

Isobaric 4-plex Tagging for Absolute Quantitation of Biological Acids in Diabetic Urine using Capillary LC-MS/MS

Michael R. Armbruster, Scott F. Grady, Christopher K. Arnatt*, James L. Edwards*

Department of Chemistry and Biochemistry, Saint Louis University, 3501 Laclede Ave, St Louis, MO 63103, United States

Contents:

Figure S1: LC-HRMS[1] analysis of each synthesized tag.

Figure S2: Proton NMR of each synthesized tag.

Figure S3: HRMS[2] analysis of tagged sarcosine.

Figure S4: PRM analysis of creatinine.

Figure S5: HRMS[2] analysis of tagged adipic acid.

Figure S6: HRMS[2] analysis of tagged pantothenic acid.

Figure S7: Minimal hexadecyl-tag is observed in the extracted samples.

Table S1: Creatinine analysis results.

Table S2: Quantified acids in control and T1D urine.

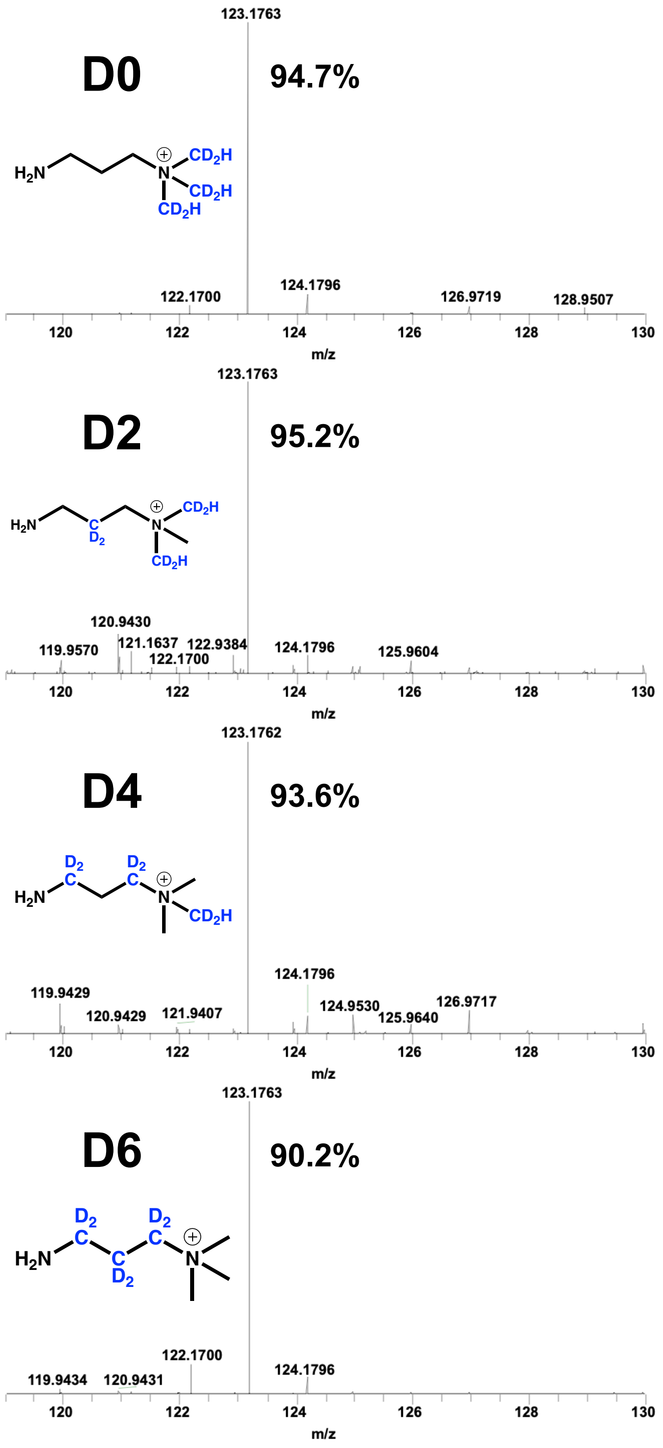

Figure S1: LC-HRMS[1] analysis of each synthesized tag.

Tags D0 – D6 were injected at 50μM for exact mass analysis. Isotopic purity is displayed to the right of each expected peak. All tags were detected within 1ppm.

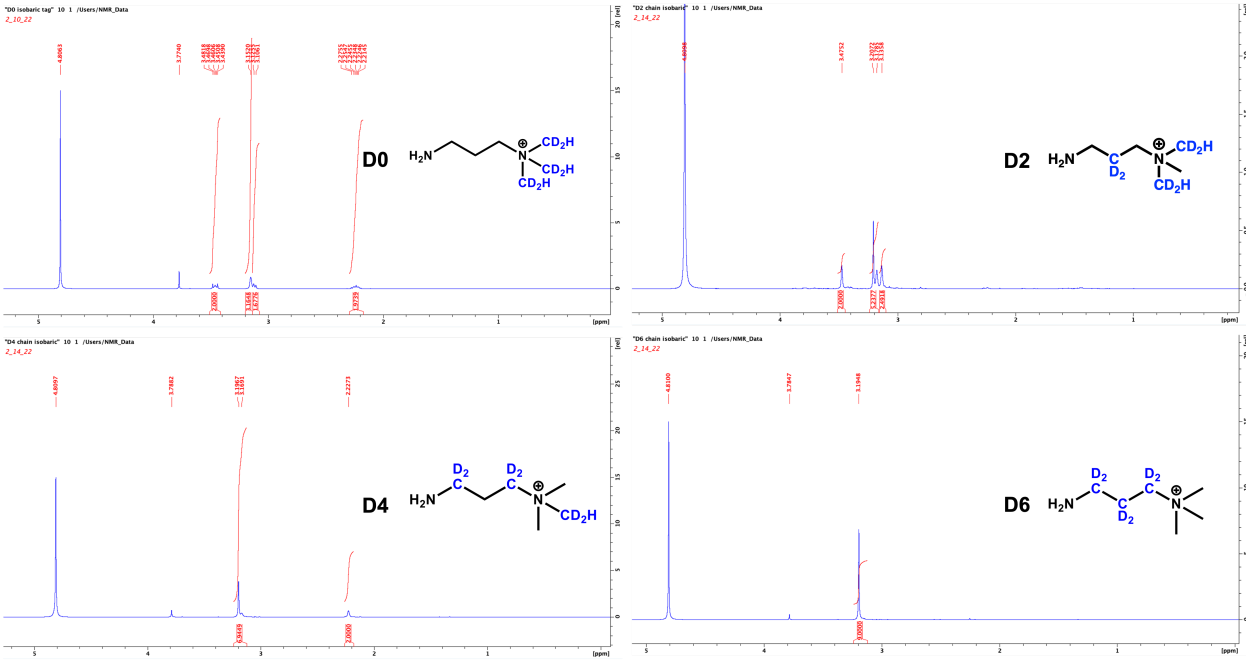

Figure S2: ^{1}H NMR of the isobaric tags.

Proton NMR was acquired at 400MHZ in D_2O. The tag structure is overlaid for each spectra.

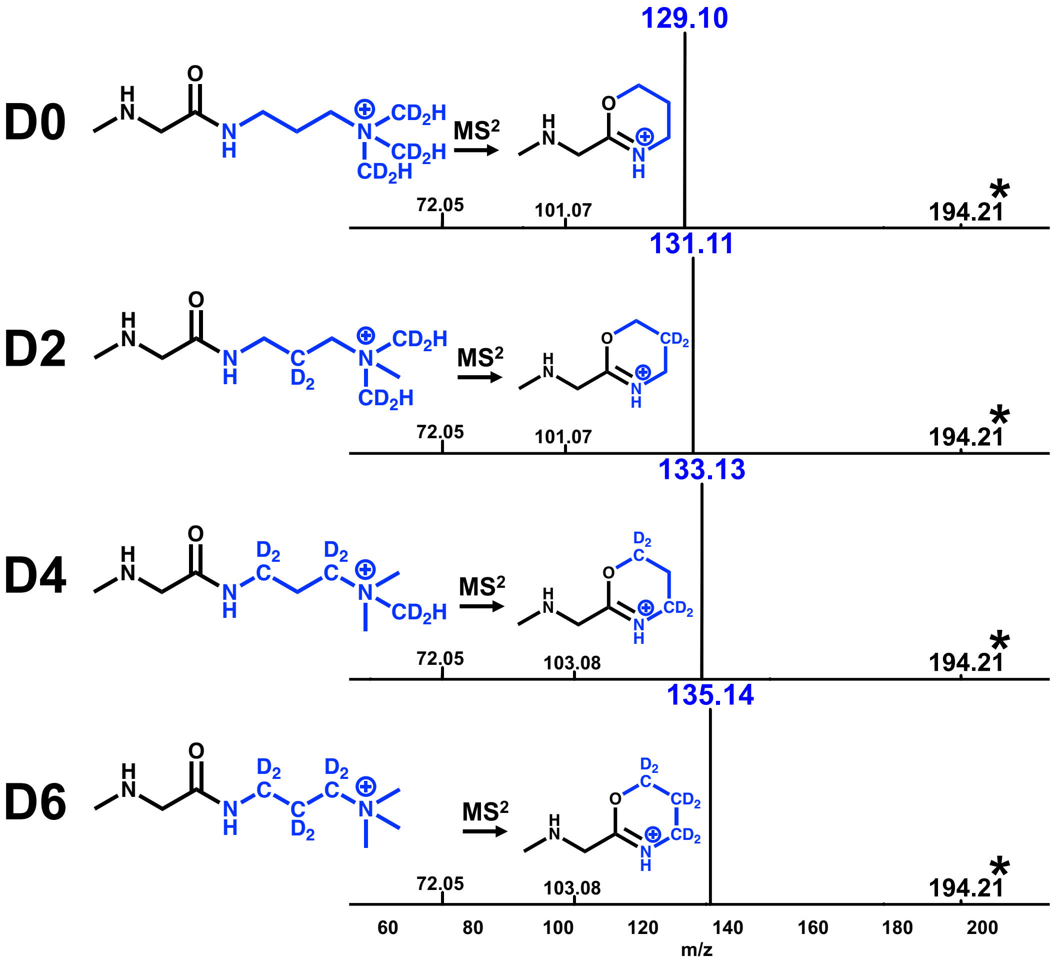

Figure S3: $HRMS^2$ analysis of tagged sarcosine.

Sarcosine was individually tagged with D0 – D6 as described in the main text. Each sample was individually injected and fragmented. Reporter ions show minimal overlap.

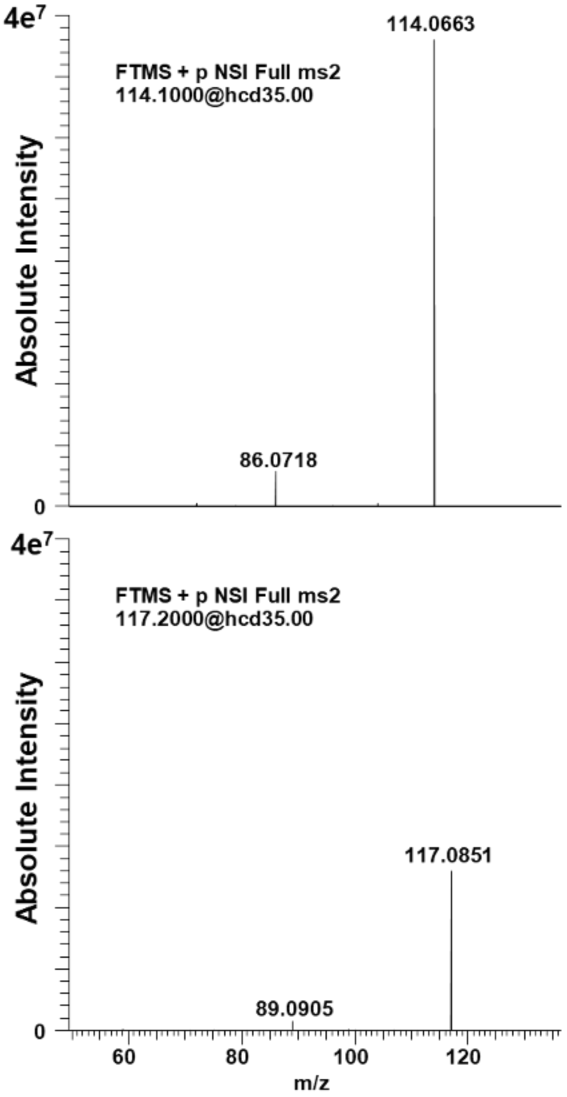

Figure S4: PRM analysis of creatinine.

Methyl-D_3 creatinine was used into each biological urine samples for the absolute quantitation of creatinine based on isotope ratio. Experimental MS^2 spectra show the biological, light creatinine (top) and D_3 labeled creatinine (bottom).

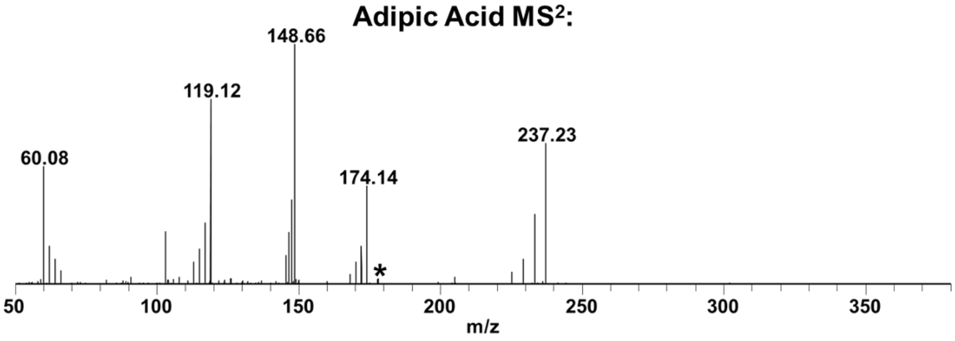

Figure S5: HRMS^2 analysis of tagged adipic acid.

Adipic acid (m/z 178.2) was reacted with the 4 isobaric tags, then mixed 1:2:5:10. Despite containing a mix of reporters from single and double ring formation, the ratios remain approximately equal. Single ring formation results in a 4 ion cluster ending with m/z 148.66, while complete ring formation produces the reporter cluster ending at m/z 119.12. Asterisk represents the precursor ion.

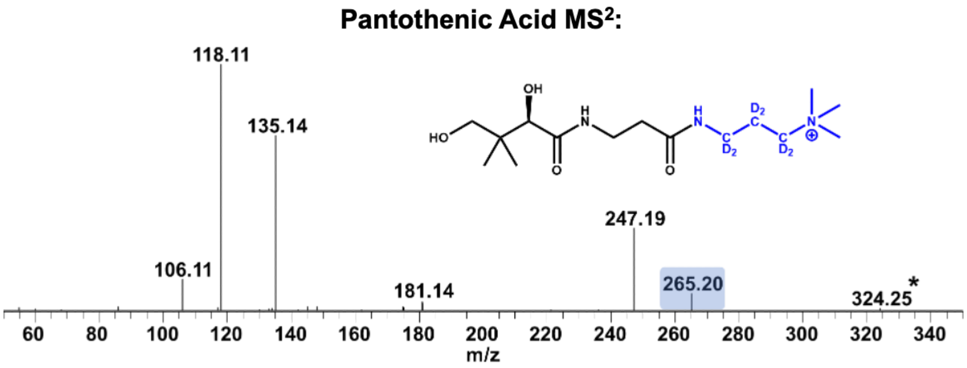

Figure S6: $HRMS^2$ analysis of tagged pantothenic acid.

Pantothenic acid was reacted with tag D6. Expected reporter based on the expected neutral loss of 59 is highlighted in blue. Acceptable reporter intensity is achieved despite an amide bond and nearby mobile proton on the native analyte.

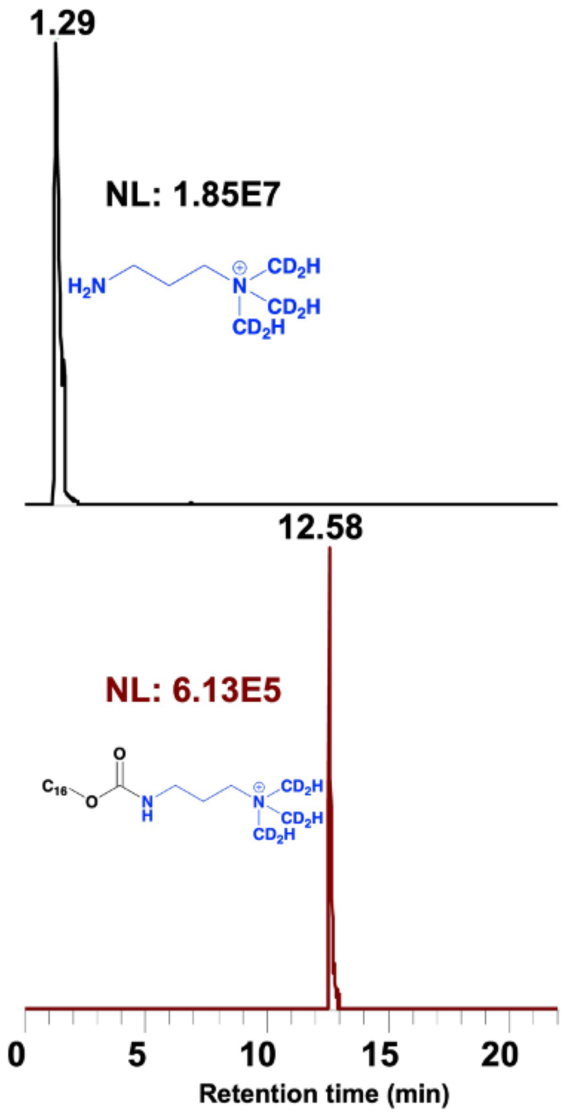

Figure S7: Minimal hexadecyl-tag is observed in the extracted samples.

Tag D0 was coupled to pooled urine as described in the main text. The sample was coupled with hexadecyl chloroformate to react with excess tag then removed using a Folch extraction. The remaining unreacted tag in the aqueous layer is approximately the intensity of one tagged analyte (top) while the coupled hexadecyl-tag is observed at low intensity (bottom).

Table S1: Creatinine analysis results.

Creatinine in urine was quantified based on the isotope ratio to methyl-D_3 creatinine using a PRM method. All samples were diluted with water to 277 μg/mL creatinine for normalization before tagging.

**Sample**	**Gender**	**Birth Year**	**Creatinine (ug/mL)**
Control 1	Male	1995	483.99
Control 2	Female	1993	704.55
Control 3	Male	1994	2191.59
T1D 1	Male	1977	3676.30
T1D 2	Male	1978	277.63
T1D 3	Male	1981	1436.98

Table S2: Quantified acids in control and T1D urine.

Control and T1D samples (n = 3 each) were tagged with D0, D4, and D6. Each sample set was mixed and injected with isotope labeled standards for each analyte. Analytes which were not detected in all six biological samples were not included.

Analyte	Ctrl μmol/mmol creatinine	Standard deviation (Ctrl)	T1D μmol/mmol creatinine	Standard deviation (T1D)	p value
3-Methyl-2-oxovaleric acid	2.56	1.87	2.46	2.25	0.957
Mevalonic acid	0.07	0.04	0.04	0.02	0.332
2-Hydroxybutyric acid	10.85	9.75	114.21	147.65	0.293
3,4-Dihydroxyphenylacetic acid	0.32	0.19	0.22	0.17	0.557
4-Acetamidobutanoic acid	6.46	1.02	4.13	1.64	0.105
4-Hydroxybenzoic acid	5.03	6.97	5.50	6.90	0.939
Adipic acid	5.37	6.47	2.87	1.75	0.553
Azelaic acid	1.54	1.05	2.36	1.07	0.397
Capric acid	0.33	0.14	0.25	0.05	0.351
Caproic acid	0.79	0.57	0.43	0.09	0.346
Caprylic acid	0.45	0.15	0.06	0.01	0.011
Dimethyl Glycine	0.29	0.15	1.52	0.73	0.046
Gluconate	13.12	8.45	6.22	6.19	0.317
Hippuric acid	149.28	149.26	374.77	278.76	0.284
Hydroxyphenyllactic acid	3.12	3.47	67.22	39.84	0.050
Lactate	3.50	2.92	6.15	1.05	0.213
Malate	2.83	2.63	7.15	4.37	0.216
Monomethyl Glutaric acid	2.36	0.68	0.32	0.21	0.008
Myristic acid	0.26	0.11	0.16	0.02	0.223
N-Acetylalanine	0.71	0.04	1.83	1.23	0.190
N-Acetylglycine	1.48	1.02	3.84	1.59	0.097
N-Acetylleucine/N-Acetylisoleucine	2.12	1.48	2.90	1.02	0.496
N-Acetyl-methionine	6.83	6.30	14.31	15.84	0.490
N-Acetylphenylalanine	0.11	0.03	0.27	0.02	0.001
N-Acetylproline	0.36	0.32	0.45	0.17	0.711
N-Acetylserine	2.15	1.26	2.21	0.94	0.950
Pantothenic acid	0.83	0.13	0.59	0.44	0.115
Pelargonic acid	1.49	1.10	1.64	0.87	0.860
p-Hydroxyphenylacetic acid	103.24	20.51	0.23	0.01	0.001
Pimelic acid	2.21	0.97	2.67	1.01	0.596
Sarcosine	0.38	0.46	0.10	0.06	0.364

Suberic acid	1.68	1.05	3.03	1.60	0.361
Ureidopropionic acid	5.40	1.03	3.91	0.42	0.082
Valeric acid	10.00	0.96	4.85	0.98	0.003
